# Deep Learning and Single‐Cell Sequencing Analyses Unveiling Key Molecular Features in the Progression of Carotid Atherosclerotic Plaque

**DOI:** 10.1111/jcmm.70220

**Published:** 2024-11-25

**Authors:** Han Zhang, Yixian Wang, Mingyu Liu, Yao Qi, Shikai Shen, Qingwei Gang, Han Jiang, Yu Lun, Jian Zhang

**Affiliations:** ^1^ Department of Vascular Surgery The First Hospital of China Medical University Shenyang Liaoning China

**Keywords:** carotid atherosclerotic plaques, deep learning, diagnostic biomarker, machine learning, single‐cell

## Abstract

Rupture of advanced carotid atherosclerotic plaques increases the risk of ischaemic stroke, which has significant global morbidity and mortality rates. However, the specific characteristics of immune cells with dysregulated function and proven biomarkers for the diagnosis of atherosclerotic plaque progression remain poorly characterised. Our study elucidated the role of immune cells and explored diagnostic biomarkers in advanced plaque progression using single‐cell RNA sequencing and high‐dimensional weighted gene co‐expression network analysis. We identified a subcluster of monocytes with significantly increased infiltration in the advanced plaques. Based on the monocyte signature and machine‐learning approaches, we accurately distinguished advanced plaques from early plaques, with an area under the curve (AUC) of 0.899 in independent external testing. Using microenvironment cell populations (MCP) counter and non‐negative matrix factorisation, we determined the association between monocyte signatures and immune cell infiltration as well as the heterogeneity of the patient. Finally, we constructed a convolutional neural network deep learning model based on gene‐immune correlation, which achieved an AUC of 0.933, a sensitivity of 92.3%, and a specificity of 87.5% in independent external testing for diagnosing advanced plaques. Our findings on unique subpopulations of monocytes that contribute to carotid plaque progression are crucial for the development of diagnostic tools for clinical diseases.

## 1 Introduction

Carotid atherosclerosis, the main cause of cerebrovascular disease, is a chronic inflammatory arterial disease characterised by the accumulation of lipids in the vessel walls, leading to the formation of atherosclerotic plaques [1]. Advanced and unstable carotid plaques pose a greater risk than early carotid plaques as they are more prone to rupture and lead to cerebrovascular events [2, 3]. Approximately 18%–25% of ischaemic strokes are caused by thromboembolism associated with carotid atherosclerotic disease [4]. Ischaemic stroke remains the second leading cause of death worldwide and the primary cause of acquired long‐term disability, resulting in high healthcare expenses [5]. Inappropriate selection or poor surgery can significantly reduce the benefits of carotid endarterectomy and stenting, which are well‐proven techniques for preventing ischaemic stroke [6]. Therefore, early screening, selection, and timely personalised management are crucial for reducing mortality and disability rates and improving outcomes. Many patients with carotid atherosclerosis are often unaware of the potential risks until the onset of symptoms, at which point unavoidable consequences have already occurred [7]. Over the past decades, numerous efforts have been made to identify biomarkers involved in the development and rupture of carotid atherosclerosis. Some markers associated with inflammation, thrombosis, and matrix degradation have been identified, the examples being interleukin (IL)‐6, C‐reactive protein, plasminogen activator inhibitor‐1, and matrix metalloproteinases (MMPs) [8, 9, 10]. Current international guidelines recommend the early identification and evaluation of risk and intervention before symptoms appear [11]. Addressing this challenge is difficult because of its complex biological and clinical characteristics [12]. Therefore, there is a pressing necessity to identify specific biomarkers that represent the advancement and stability of carotid plaques for their early detection and timely personalised treatment.

The progression of early‐stage lesions to advanced‐stage lesions is influenced not only by inflammation, oxidative stress, and lipoprotein aggregation [13, 14], but also by physical, haemodynamic, and biomechanical factors [15]. Immune and inflammatory responses have been shown to play essential roles in the progression of atherosclerotic plaques [16]. Immune cells, such as T and B cells and macrophages, infiltrate aortic wall lesions during carotid atherosclerosis [17]. These activated immune cells secrete various inflammatory factors such as IL‐1β, IL‐6, and (tumor necrosis factor (TNF)‐α, which facilitate plaque development [18]. However, the development and progression of carotid atherosclerosis are influenced by multiple factors, and many unknown pathological mechanisms require further investigation. No reports have identified the leading molecular processes and their interactions with inflammation‐related pathways that govern the progression of carotid atherosclerotic plaques (CAS). Therefore, the key markers and pathological mechanisms of carotid atherosclerosis progression must be further investigated to develop and improve personalised management strategies.

Advancements in high‐throughput sequencing technologies, such as microarrays, RNA‐sequencing (RNA‐seq), and single‐cell RNA‐sequencing (scRNA‐seq), have facilitated the investigation of specific immune cell populations and key biomarkers associated with multiple diseases. These findings have great potential for the screening, diagnosis, and management of diseases [19, 20]. Machine learning is widely used as a valuable tool to essential important cell types and identify key biomarkers by efficiently analysing high‐dimensional sequencing data using unique downscaling and classification algorithms [21]. With the progress of artificial intelligence methods, deep learning models have recently gained popularity in genomic and epigenomic studies, owing to their capabilities in discovering intricate structures in large‐scale high‐throughput bioinformatics data, allowing for highly accurate prediction and classification. This level of accuracy is challenging to achieve using conventional machine‐learning algorithms [22, 23]. In the current study, we identified key biomarker genes and specific immune cell types in advanced carotid atherosclerosis using comprehensive bioinformatics approaches and machine‐learning strategies. Additionally, a novel carotid atherosclerosis progression prediction model was established using machine and deep learning methods. Novel biomarkers provide new perspectives for the disease prediction, diagnosis, targeted prevention, and personalised management of carotid atherosclerosis.

## 2 Materials and Methods

### 2.1 scRNA‐Seq Acquisition and Processing

The scRNA‐seq dataset GSE159677 consists of three samples of atherosclerotic core (AC) plaques and three samples of patient‐matched proximal adjacent (PA) portions of the carotid artery sourced from the GEO dataset [24]. The “Seurat” package in R studio was used to process and quality control the scRNA‐seq data. Cells that expressed between 200 and 2500 genes, with < 10% of mitochondrial genes expressed, were retained. The ‘FindVariableFeature’ function was used to identify 2000 highly variable genes in each sample, while the “Harmony” package was used to eliminate the batch effects. Cell clusters were obtained using the “FindClusters” method and presented using the uniform manifold approximation and projection (UMAP). The cell types were annotated using SingleR software. Then, proofreading was performed based on the expression of known markers, as shown in Data S1. To further identify specific subpopulations of monocytes in advanced carotid plaques, monocytes were re‐dimensionalized and re‐clustered.

### 2.2 Cell–Cell Communication Analysis

First, we selected a subcluster specific to the AC based on the results of the clustering of monocytes and named them “atherosclerotic core‐associated monocytes” (AC mono). The remaining monocyte subclusters were referred to as “other monocytes” (Other mono). Genes with log2FoldChange absolute values > 1 and adjusted *p* value < 0.05 were identified as differentially expressed genes between AC mono and Other mono using the ‘FindAllMarkers’ method in R package “Seurat”. We then used the CellChat package to visualise the intercellular interactions mediated by ligand–receptor complexes [25]. Ligand–receptor interactions in the CellChat database for humans (CellChatDB.human) were used to assess the main signal inputs and outputs in all monocyte subclusters.

### 2.3 Co‐Expression Network Analysis of Single‐Cell Transcriptomic Data

High‐dimensional weighted gene co‐expression network analysis (hdWGCNA) (https://smorabit.github.io/hdWGCNA/reference/index.html) is a WGCNA analysis package used for single‐cell transcriptome data. The monocyte clusters were selected as “Seurat” objects for further analysis. Single‐cell sequencing data for WGCNA analysis were processed using the hdWGCNA package following the standard procedure [26, 27]. The results were presented as monocyte–subcluster UMAP plots and dot plots. Modules highly associated with AC mono were defined as hub modules.

### 2.4 Pseudotime Trajectory Analysis of Monocyte Cluster

The “monocle” package was used to analyse the differentiation trajectories of the extracted monocyte cluster. After dimensionality reduction using the “DDRTree” method, the plot_cell_trajectory function was used to visualise the differentiation trajectories of the cells.

### 2.5 Functional Enrichment Analysis

We selected the first 25 genes from each hub module, resulting in a total of 100 hub module genes for enrichment analysis. Metascape (http://metascape.org) was used to analyse the biological functions and pathways of these genes through Gene Ontology (GO) [28], Kyoto Encyclopaedia of Genes and Genomes [29], and Reactome [30] enrichment analyses. We assessed the metabolism score of different monocyte subclusters by obtaining gene sets involved in metabolism from the Molecular Signatures Database (MSigDB). We then calculated the scores using the “AddmoduleScore” function and visualised the results of interest using a violin plot.

### 2.6 Bulk RNA‐Seq Acquisition and Processing

The GSE163154, GSE28829, and GSE43292 datasets were acquired using the R package GEOquery. After data annotation, normalisation, and batch correction, GSE163154 was used as the training set for nonnegative matrix factorization (NMF) classification, machine learning, and deep learning diagnostic model construction. GSE28829 was used as an external test dataset to validate the diagnostic performance of the model, whereas GSE43292 was used to verify the expression levels of hub genes.

### 2.7 Least Absolute Shrinkage and Selection Operator (LASSO) Regression and Multiple Integrated Machine Learning

We initially used single‐factor logistic regression to screen hub module genes and selected those with discriminatory values for subsequent analysis. Then, the LASSO algorithm, via the R package “glmnet”, was used to determine hub diagnostic genes. Mlr3verse, an R package that integrates various machine‐learning algorithms, was used to assess the diagnostic performance of LASSO genes to differentiate between patients with early‐ and advanced‐stage CAS. The machine‐learning method with the most accurate diagnostic results was selected for external testing.

### 2.8 Assessment of Differences in Enrichment Scores of Hub Module Genes and Characterisation of Molecular Subtypes

We quantified the enrichment results of hub module genes in the GSE163154 dataset using the “GSVA” package [31]. Then “limma” package was used to analyse the differences in gene set variation analysis (GSVA) results between advanced‐stage plaque and early‐stage plaque samples. Advanced plaque samples were re‐clustered according to hub module genes using the NMF clustering algorithm. GSVA was performed for the different subtypes.

### 2.9 Assessment of Immune Cell Infiltration in Subtypes and Its Correlation With Hub Genes

The Immuno‐Oncology Biological Research (IOBR) R package was used to analyse immune features and immune cell infiltration in different subtypes. Spearman's correlation coefficients were calculated for immune features with the LASSO genes.

### 2.10 Construction of Convolutional Neural Networks for Deep Learning Disease Prediction Models

Using the “IOBR::deconvo_mcpcounter” function, 10 types of immune cells in the test data set GSE163154 and training data set GSE28829 samples were evaluated. The immune cell composition data were then merged with the gene expression data to generate a heat map. This heat map is a “portrait” of each patient, created based on the ratio of expression levels of central genes to expression levels of immune cells. This step converts complex gene expression patterns into a format suitable for input to deep learning algorithms. A convolutional neural network (CNN) deep learning model was built using the “keras” package. Briefly, our CNN model contains two convolutional layers. The former used 32 filters with a 3 × 3 convolution kernel, while the latter had 16 filters and a 2 × 2 convolution kernel size. A max‐pooling layer using a 2 × 2 window size was then added, followed by flattened layers and fully connected layers. Finally, the model was set up using the Adam optimizer, with binary cross‐entropy as the loss function and accuracy used as the evaluation metric. The training process was repeated 500 times. More details about the CNN model can be found in the Data S1.

### 2.11 Clinical Sample Collection

Carotid atherosclerotic plaques were collected from the patients who underwent carotid endarterectomy. This research adhered to the principles outlined in the Declaration of Helsinki. Approval was granted by the Ethics Committee of the First Hospital of China Medical University (Ethics approval number: 2024‐573). All participants received comprehensive information about the study and signed informed consent forms.

### 2.12 Cell Culture and Treatment

Raw264.7 cells were purchased from Procell (CL‐0190, China) and cultured in specialised culture media (CM‐0190; Procell, China). The cells were then incubated at 37°C and 5% CO_2_. Cells were inoculated into six‐well plates at a density of 1.0 × 10^6^/mL per well and treated with 100 ng/mL LPS (SIGMA) for 24 h to activate them into M1 macrophages.

### 2.13 Quantitative Real‐Time Polymerase Chain Reaction (qRT‐PCR)

Total RNA was isolated from macrophages and plaques using RNAkey Total RNA Extraction Reagent (Sevenbio, China), following the manufacturer's guideline. Subsequently, cDNA was produced by reverse transcription of RNA using a Premix Pro TaqHS qPCR kit (Accurate Biotechnology, AG11718). The primer sequences used are provided in Data S1. The primers used for this study were synthesised by BGI TECH (Beijing, China). β‐Actin was for use as an internal reference gene and 2−ΔΔCt process was used to determine the expression levels of the target genes.

### 2.14 Histological, Immunofluorescence and Immunohistochemical Analyses

Six fresh CAS specimens were divided into advanced‐stage (AC plaques) and early‐stage plaques (patient‐matched PA plaques). Samples were fixed overnight in 4% neutral formaldehyde and buried in paraffin. Serial sections (5 mm thick) were cut from the samples. The slides were de‐paraffinised and antigenically repaired. Immunoreactions were executed according to the suggested protocols of the UltraSensitive SP (Mouse/Rabbit) immunohistochemistry (IHC) Kit (MXB Biotechnologies, KIT‐9730). The following primary antibody was used: anti‐FTH1 (1:200, A1144, Abclonal). Additionally, we used integrated optical density measuring by Image‐Pro Plus 6.0 software to quantify the IHC results.

Regarding the immunofluorescence staining of sections from carotid plaque tissues, the procedures before antigenically repaired were the same as the IHC. After blocked with blocking solution (Beyotime, P0260) for 30 min, sections were incubated with primary rabbit antibodies against FTH1(1:50, Abclonal) and mouse antibodies against CD68 (1:50, ab955, Abcam) overnight at 4°C. The corresponding secondary antibodies were used for incubating for 1 h at room temperature in darkness, including Goat Anti‐Rabbit IgG H&L (1:500, ab150080, abcam) and Goat Anti‐Mouse IgG H&L (1:500, ab150113, abcam). Nuclei were stained with 4',6‐diamidino‐2‐phenylindole (DAPI) (Solarbio, C0065). Finally, images were visualised and photographed by a fluorescence microscope (Evos FL Auto, Thermo Fisher, USA).

### 2.15 Statistical Analysis Methodology

All data processing, statistical analysis, and plotting were performed using R version 4.1.3. Statistical significance was compared between the two groups using Student's *t*‐test or Wilcoxon rank‐sum test. The Shapiro–Wilk test was used to verify the normality of the data to decide whether to use the Student's *t*‐test. If the data met the assumption of normality, we used the Student's *t*‐test. If the data failed to meet the requirements of normal distribution, or if the sample size was too limited to appropriately perform the *t*‐test, we selected the Wilcoxon rank‐sum test as a non‐parametric alternative method. This ensures that our statistical analysis is reliable and valid [32]. Correlations between variables were assessed using Pearson's correlation coefficient. Statistical analysis between each cell type and between each cluster of monocytes was performed using Fisher's exact test and post‐hoc function in RStudio was used to adjust *p* values. *p*‐value < 0.05 was regarded as statistically significant.

## 3 Results

### 3.1 Single‐Cell Profiling of Early and Advanced Atherosclerotic Plaques in Carotid Artery

**FIGURE 1:**
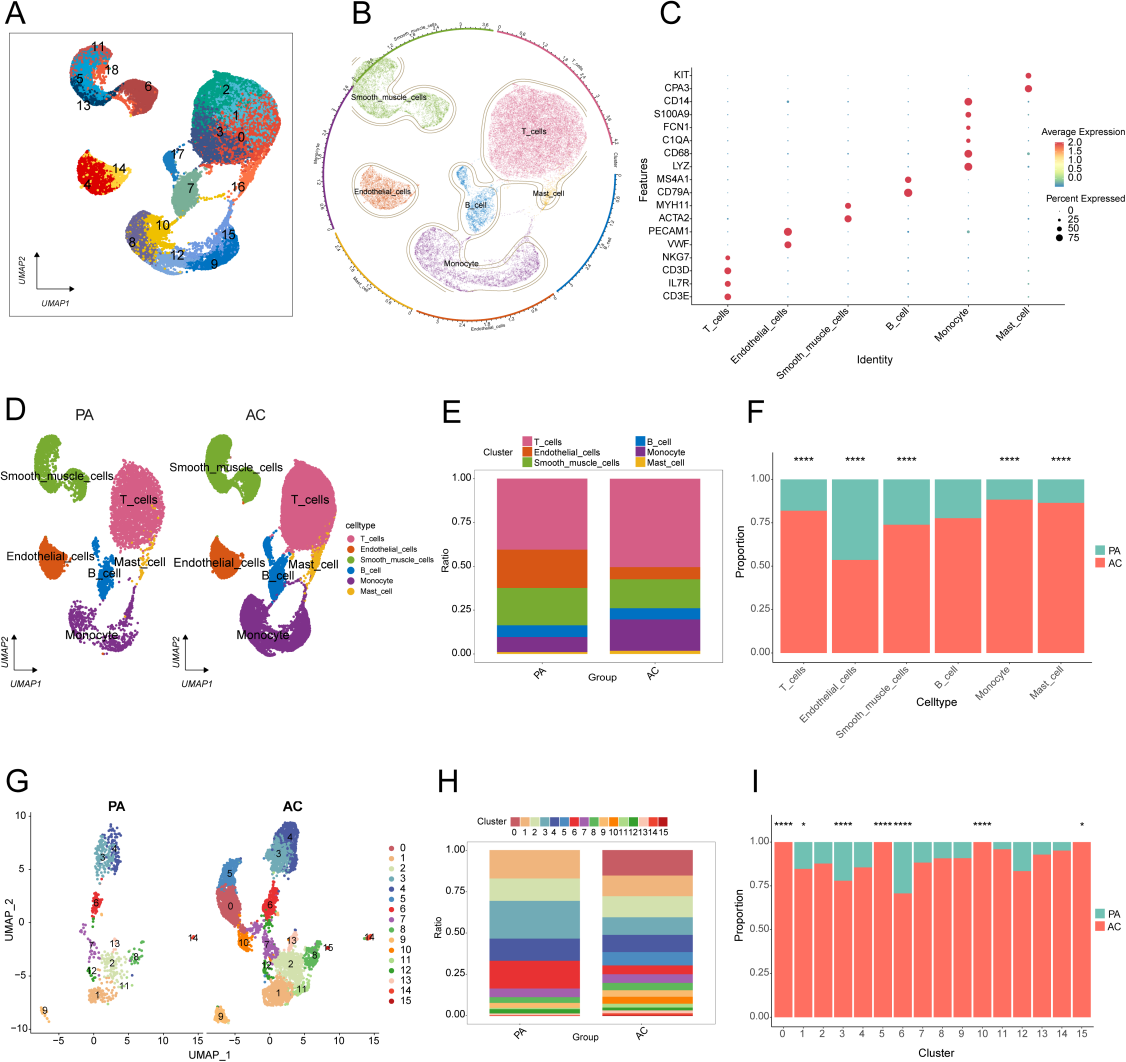
Single‐cell profiling of early and advanced carotid atherosclerotic plaques. (A) UMAP dimensionality reduction includes 19 clusters. (B) UMAP plot displaying the annotation of six cell types in the carotid plaques. (C) The expression of representative marker genes in each cluster in a dot plot. (D) UMAP plot displaying the increased proportion of monocyte between early and advanced carotid plaques. (E) Proportion of each cell type. (F) Bar chart showing differences in proportions of six cell types between early and advanced carotid plaques. (G) UMAP plot showing altered proportions of monocyte subclusters between early and advanced carotid plaques. (H) Proportions of each monocyte cluster. (I) Bar chart showing differences in proportions of 16 monocyte subcluster between early and advanced carotid plaques. Statistical analysis was performed using Fisher's exact test and post‐hoc function in RStudio was used to adjust *p* values. ^****^ *p* < 0.0001, ***p < 0.001, *p < 0.05.

All samples in GSE159677 included three AC plaques as the experimental group and three patient‐matched PA portions of the carotid artery tissue from the same patient as the control group. After applying the previously described filtering method, 19 clusters were visualised using UMAP (Figure 1A).

These clusters were further annotated into six cell clusters using the ‘SingleR’ packages, including monocyte, B cell, T cell, smooth muscle cell (SMC), endothelial cell (EC), and mast cell (Figure 1B). Based on the known marker genes, we calibrated the annotated results and displayed the expression of marker genes in each cluster in a dot plot (Figure 1C). UMAP plots and cell scale plots were created to visually observe the differences in the cell cluster composition between the two groups (Figure 1D,E).

### 3.2 Identification of Hub Subclusters Involved in Carotid Plaque Progression

According to the results shown in the cell ratio graph, the percentage of monocytes in the AC was distinctly higher than that in the PA (Figure 1F), which may indicate that monocytes play an imperative role in the advancement of CAS. We extracted monocytes, and 15 subclusters were visualised using UMAP (Figure 1G). According to the plot of the cell subpopulation proportions between the two groups (Figure 1H), we noted that four monocyte subclusters (0, 5, 10, and 15) were specific to AC (Figure 1I). We named these four monocyte subclusters AC mono, whereas the other subclusters were named Other mono.

### 3.3 Cell–Cell Communication Analysis

**FIGURE 2:**
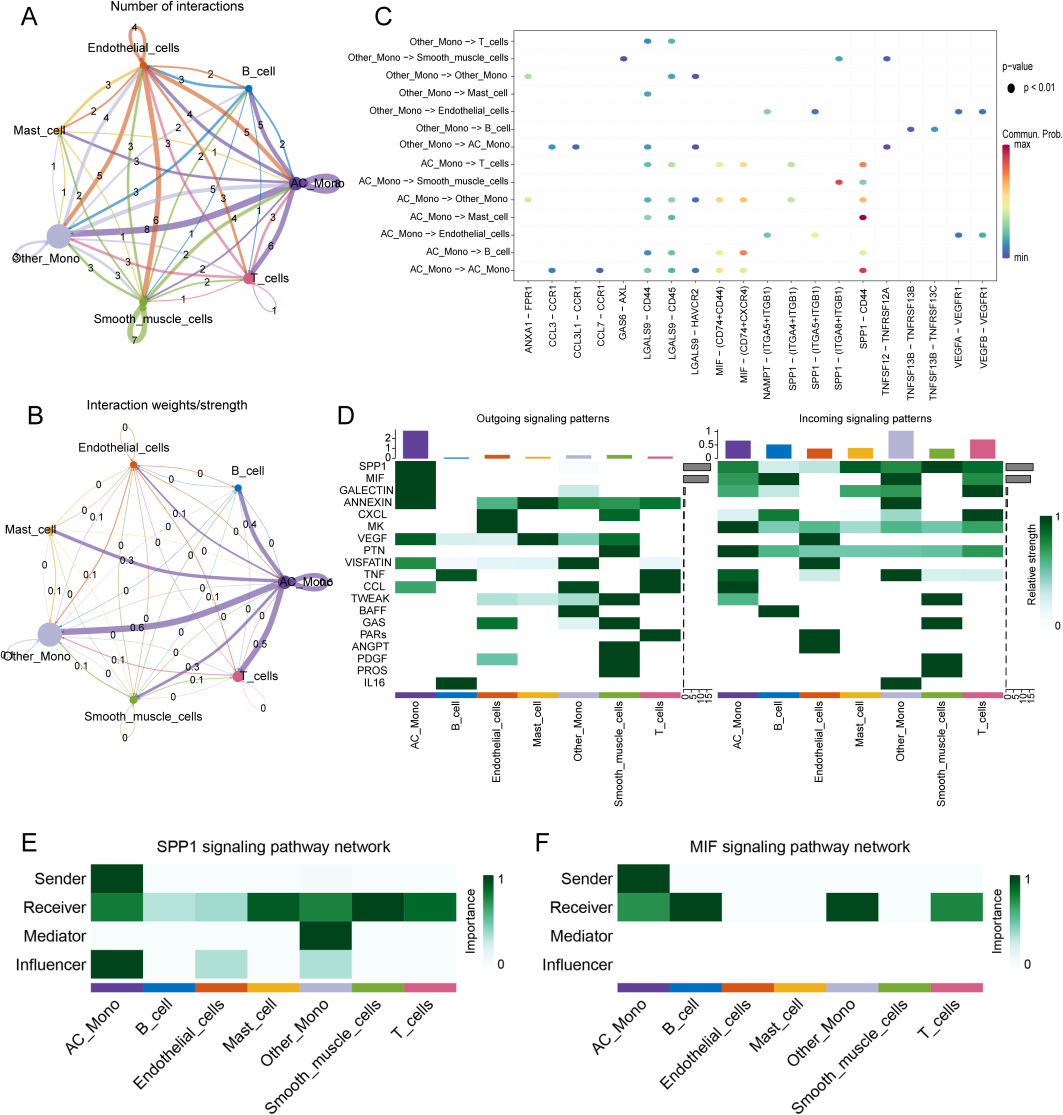
Cell–cell communication analysis. (A) Number of signals for different cell clusters in advanced plaques. (B) Intensity of the signals for different cell clusters in the advanced plaque. (C) Cell–cell interactions between monocytes and other cell types. (D) Relative strength of the outgoing and incoming signals enriched in clusters from advanced plaques. (E, F) Advanced plaque monocytes act as senders of SPP1 and MIF signalling.

Interestingly, the AC mono demonstrated more intercellular interactions with other cells than the other mono. This could be attributed to the higher level of cellular function of AC mono, which is consistent with its involvement in carotid plaque progression (Figure 2A,B). We further visualised cellular communication between AC mono and other cells because of their high degree of communication (Figure 2C,D). Based on the visualisation results, AC mono primarily functioned as a signal sender through SPP1 (Figure 2E) and MIF signalling (Figure 2F) pathway to regulate the function of other cells.

### 3.4 Construction of a Co‐Expression Network and Hub Module Identification

**FIGURE 3:**
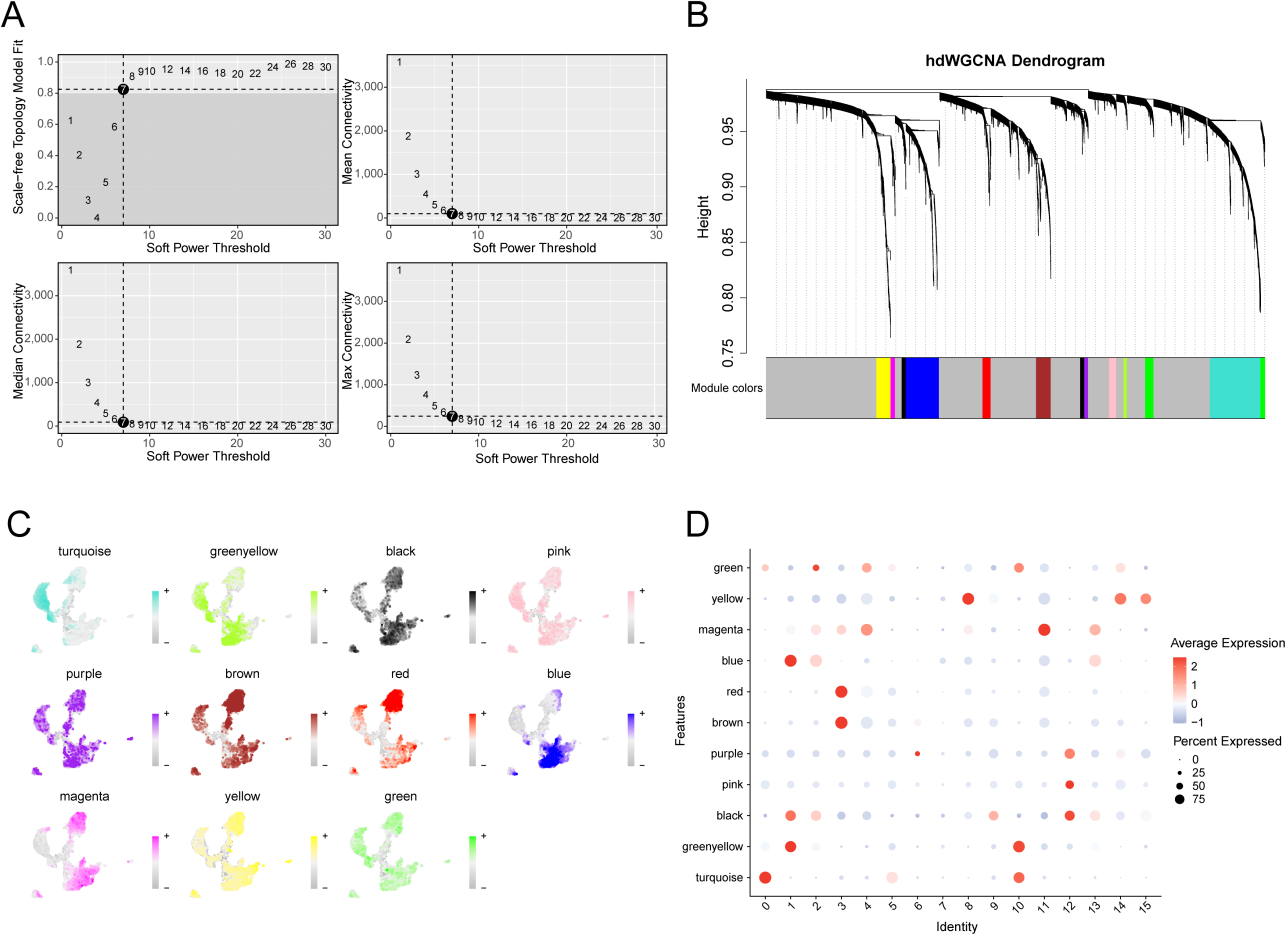
hdWGCNA revealed the hub module. (A) Optimal soft threshold equal to 7 when the network reached a scale‐free distribution. (B) hdWGCNA clustered the highly variable genes in 11 modules. (C) FeaturePlots presenting the distinct module scores in monocytes. (D) Dot plot showing the scores of separate modules in monocytes.

We selected seven as the optimal soft threshold to construct the co‐expression network and obtained 11 modules (Figure 3A,B). The feature plot and dot plot results indicated that the turquoise, green‐yellow, yellow, and green modules were more likely to be associated with the AC mono (Figure 3C,D).

### 3.5 Pseudotime Analysis of Monocytes and Functional Enrichment Analysis

**FIGURE 4:**
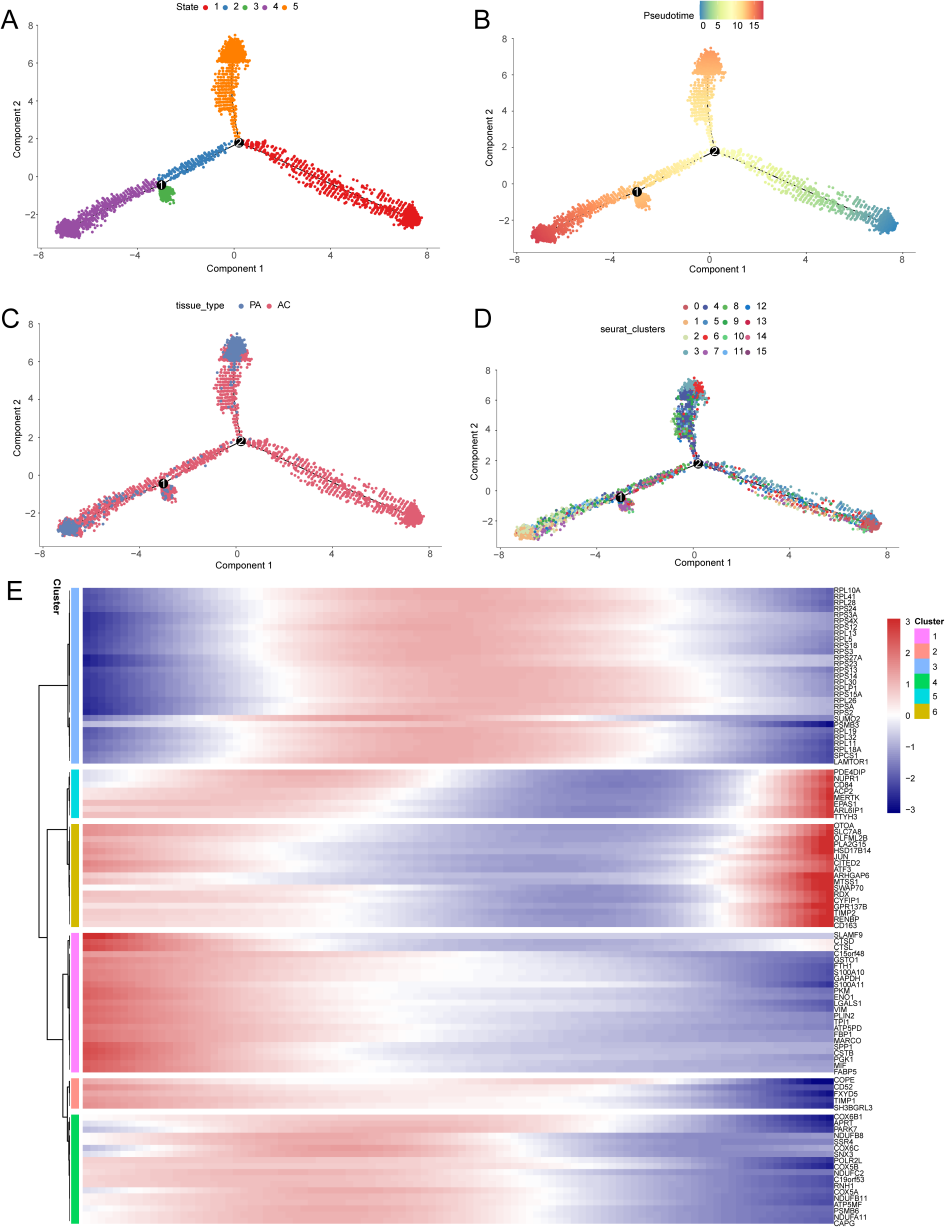
Trajectory analysis of monocytes. (A) The developmental trajectory of monocytes, colour‐coded by state (left) and Pseudotime (right). (B) The developmental trajectory of monocytes, colour‐coded by cell type (left) and cluster (right). (C) Trajectory heatmap constructed using the top 25 genes in each hub module.

To further understand monocyte dynamics, pseudotime developmental trajectory analysis was conducted. The lineage structure of monocytes in the CAS milieu was determined by analysing the developmental trajectory, which provided a distinctive picture (Figure 4A). The trajectory of monocytes showed that monocytes extracted from PA were scattered at the end of the trajectory. Cluster 0 of AC mono was primarily distributed at the start of the trajectory; clusters 5, 10, and 15 were distributed not only at the beginning but also at the end and along the trajectory (Figure 4B). We used the top 25 genes from each hub module to construct the pseudotime heatmap (Figure 4C).

**FIGURE 5:**
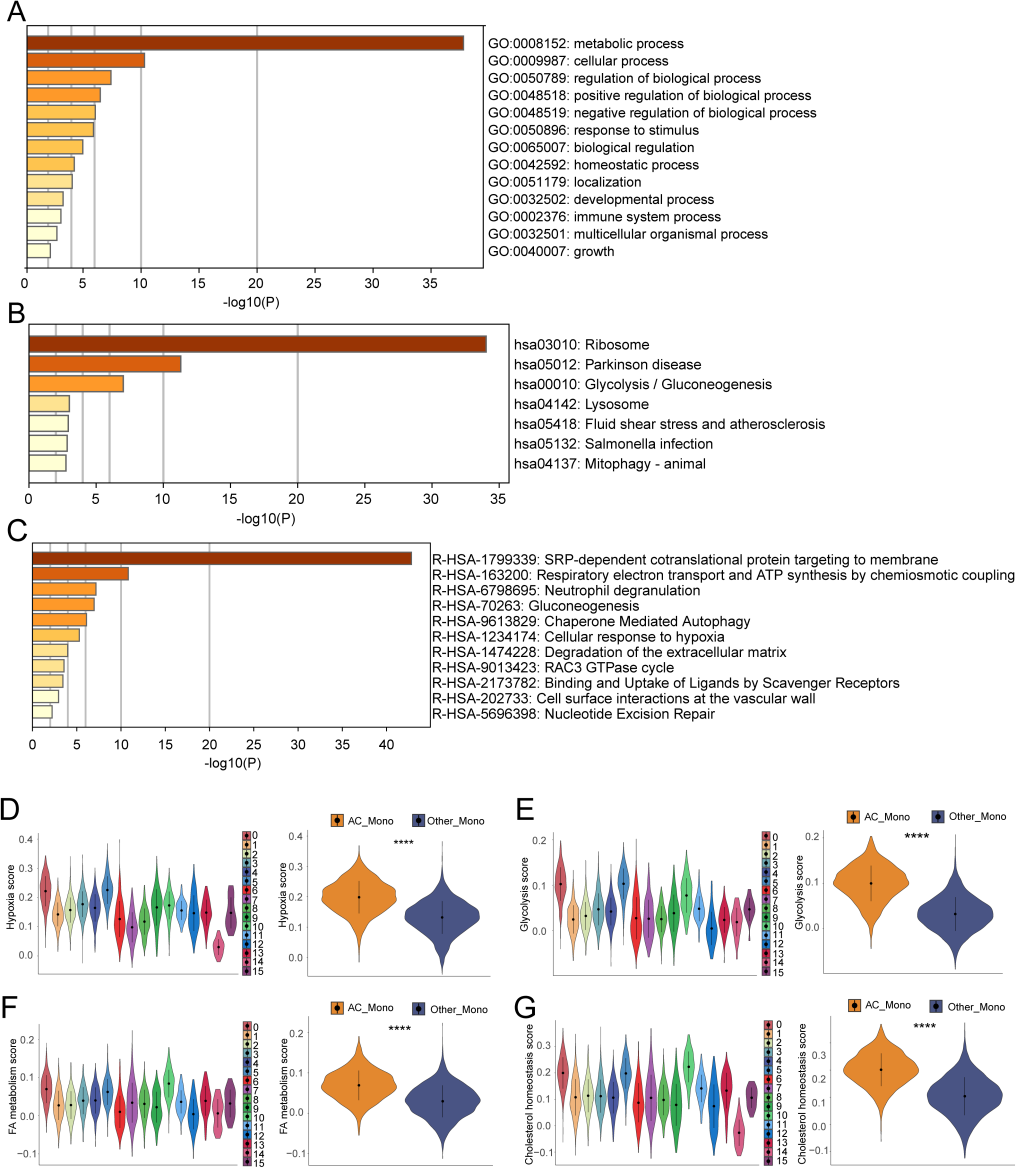
Functional enrichment analysis. (A–C) GO, KEGG, and Reactome enrichment analyses of hub module genes, coloured by *p*‐value. (D–G) Hub clusters had higher metabolic scores in hypoxia, glycolysis, fatty acid (FA) metabolism, and cholesterol homeostasis. The Wilcoxon test is employed to compare AC mono and other mono. ^****^ *p* < 0.0001.

The results revealed that AC mono were predominantly early‐stage monocytes, which possessed stronger pathogenic potency and contributed to plaque instability. Through functional enrichment analysis of the hub module genes, we observed that AC mono was highly enriched in metabolic processes (Figure 5A–C). We further found that AC mono exhibited higher metabolic scores in various areas including hypoxia, glycolysis, cholesterol homeostasis, and fatty acid metabolism (Figure 5D–G). These results indicated that alterations in cellular metabolic processes affect monocyte function, contributing to the development of carotid plaques.

### 3.6 Identification of Hub Genes and Construction of Disease Diagnostic Models Using Machine‐Learning Methods

**FIGURE 6:**
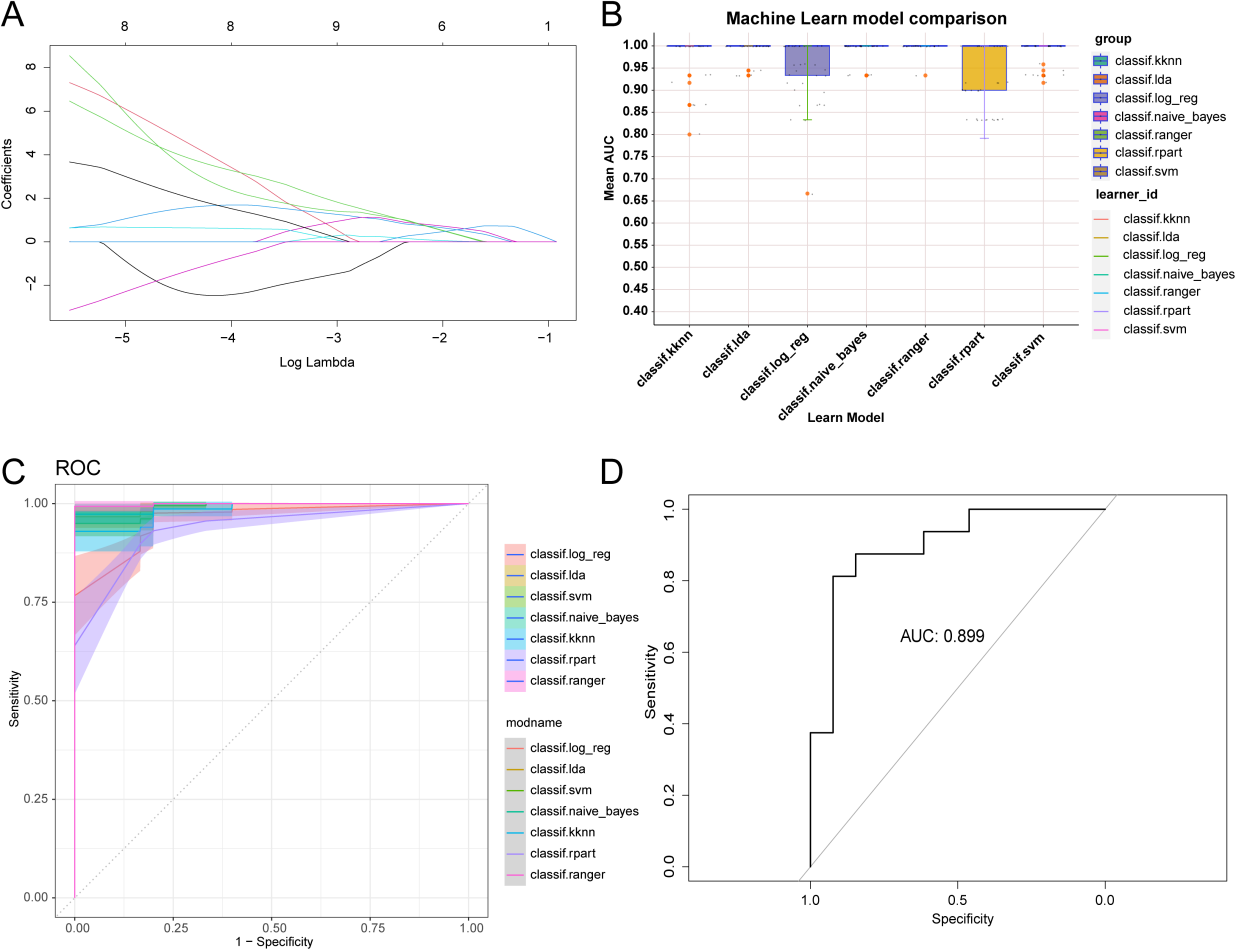
Detection of hub genes and construction of diagnostic models. (A) LASSO regression scales down genes to 8. (B) Mean AUC of 5‐fold cross‐validation for ten replications of each model. (C) ROC curves and confidence intervals for different models. (D) ROC curve for random forest model on independent external validation set.

A total of 100 genes were detected by selecting the top 25 genes ranked by kMEs of each hub module. After performing LASSO analysis, 8 hub genes were identified including Parkinsonism Associated Deglycase (*PARK7*), Ribosomal Protein S24 (*RPS24*), Ferritin Heavy Chain 1 (*FTH1*), Solute Carrier Family 7 Member 8 (*SLC7A8*), Cytoplasmic FMR1 Interacting Protein 1 (*CYFIP1*), Olfactomedin Like 2B (*OLFML2B*), Hydroxysteroid 17‐Beta Dehydrogenase 14 (*HSD17B14*), and Tissue Inhibitor of Metalloproteinase *2* (*TIMP2*) (Figure 6A). Subsequently, hub genes were used to construct multiple machine‐learning models in both the training and validation cohorts to determine their effectiveness in distinguishing advanced plaque from early plaque. Based on our findings, random forest (RF) was more accurate (Figure 6B,C). Therefore, RF was selected for external testing and an AUC value of 0.899 was achieved (Figure 6D).

### 3.7 Assessing Differences in Enrichment Scores of Hub Module Genes and Identification of Molecular Subtypes

**FIGURE 7:**
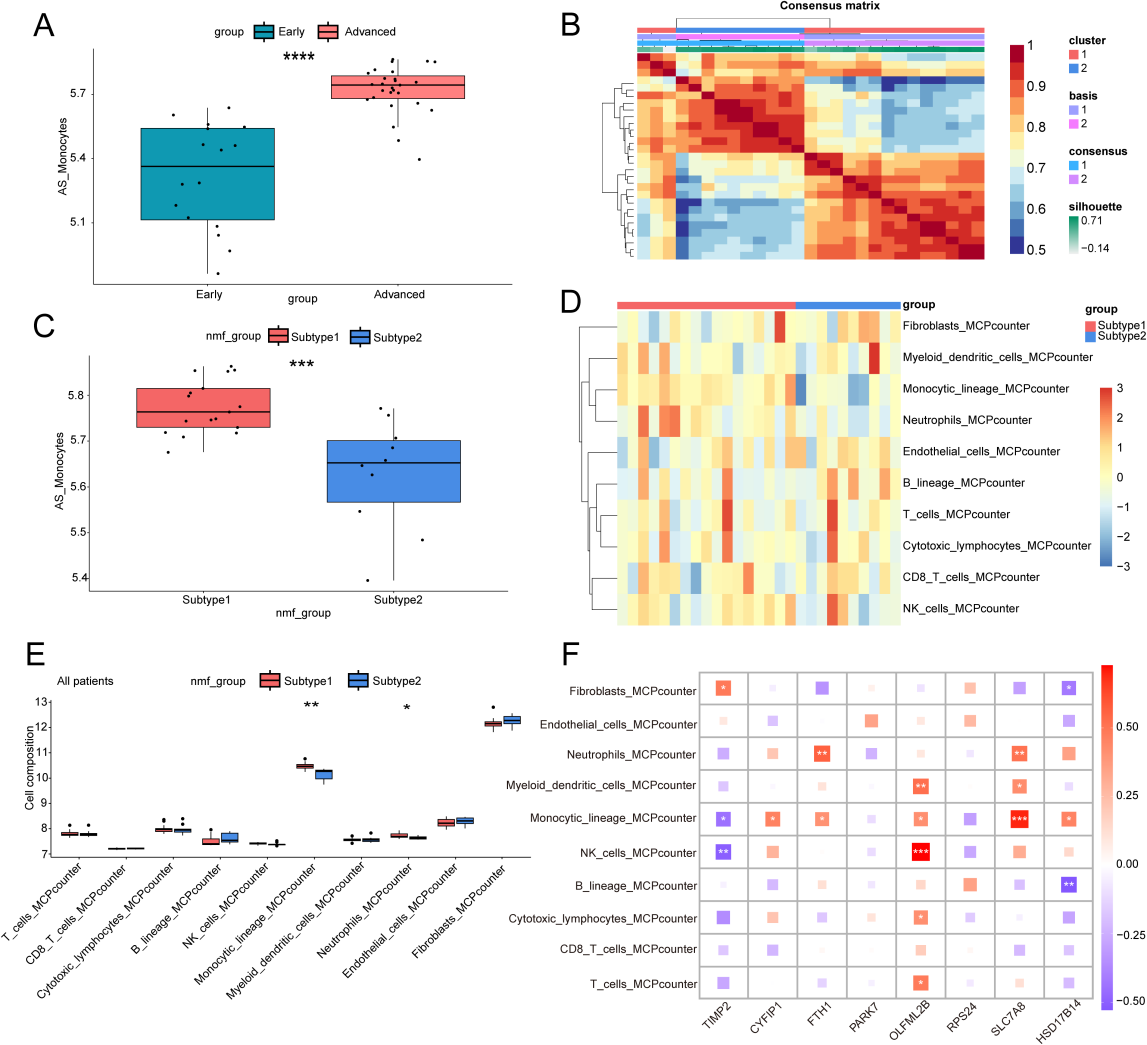
Hub module genes are relevant to immune infiltration and are available for advanced‐stage carotid plaque patient subtyping. (A) Patients with advanced carotid plaques have higher hub module scores in independent datasets. The Wilcoxon test is employed to compare the two data sets. ^****^ *p* < 0.0001. (B) NMF methods subclassified patients into two subtypes. (C) Hub module scores for the two subtypes. The Wilcoxon test is employed to compare the two data sets. ***p < 0.001. (D) Heatmap of the immune infiltration abundance between the two subtypes. (E) Variation in the abundance of immune cell infiltration between the two subtypes in the box plot. The Wilcoxon test is employed to compare the two data sets. *p < 0.05; **p < 0.01. (F) Correlation between LASSO genes and immune cells is revealed by the Pearson correlation analysis. *p < 0.05; ***p < 0.001; ^****^ *p* < 0.0001.

Using the GSVA method, we found higher monocyte scores in advanced plaques, suggesting significant involvement of monocytes in advanced carotid plaques (Figure 7A). Based on the results of hub gene identification, advanced plaque samples were divided into two subtypes (Figure 7B). The monocyte scores were higher in subtype 1 than in subtype 2 (Figure 7C).

### 3.8 Assessment of Immune Cell Infiltration in Subtypes and Its Correlation With Hub Genes

The results of immune infiltration of subtype 1 and 2 NMF clusters showed that the monocytic lineage and neutrophils were significantly upregulated in subtype 1, which differed significantly from subtype 2 (Figure 7D,E). Thereafter, relevance analysis was performed between the hub genes and immune cells, which demonstrated a positive correlation between the expression of OLFML2B and the levels of natural killer (NK) cells, myeloid dendritic cells, monocytic lineage, cytotoxic lymphocytes, and T cells. In contrast, the infiltration of myeloid dendritic cells, monocytic lineage cells, and neutrophils had a positive correlation with *SLC7A8* expression. Furthermore, infiltration of the monocytic lineage and NK cells was negatively related to *TIMP2* expression. The expression of *CYFIP1*, *FTH1*, *and HSD17B14* was positively correlated with the infiltration of the monocytic lineage (Figure 7F).

### 3.9 Construction of CNNs for Deep‐Learning Disease Diagnostic Models

**FIGURE 8:**
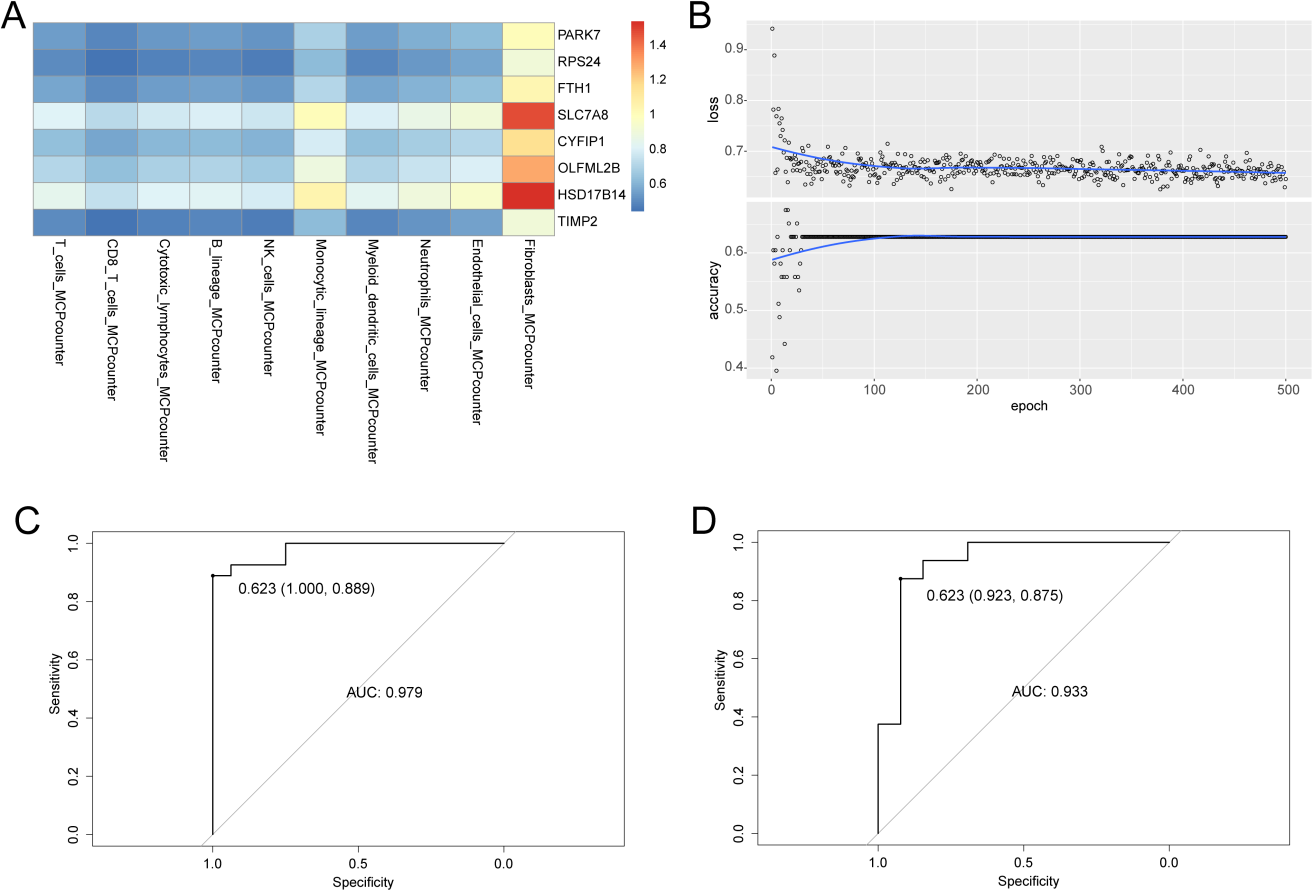
Construction of the convolutional neural network (CNN) model. (A) Gene‐immune profile of the patient used for constructing the CNN model. (B) Training procedure of CNN model. (C) Performance of the CNN model while training. (D) Performance of the CNN model while validating.

We constructed a gene‐immune heatmap for each patient, with the value of each square representing the ratio of gene expression to immune cell infiltration (Figure 8A). After obtaining a “portrait” of each patient, we performed 500 model training sessions (Figure 8B). The receiver operating characteristic (ROC) curve was used to determine the performance of the model in the training and testing datasets. The AUC value for the training dataset was 0.979, and the AUC value for the test dataset was 0.933 when the threshold value was 0.623 (Figure 8C,D).

### 3.10 Verification of the Marker Genes

**FIGURE 9:**
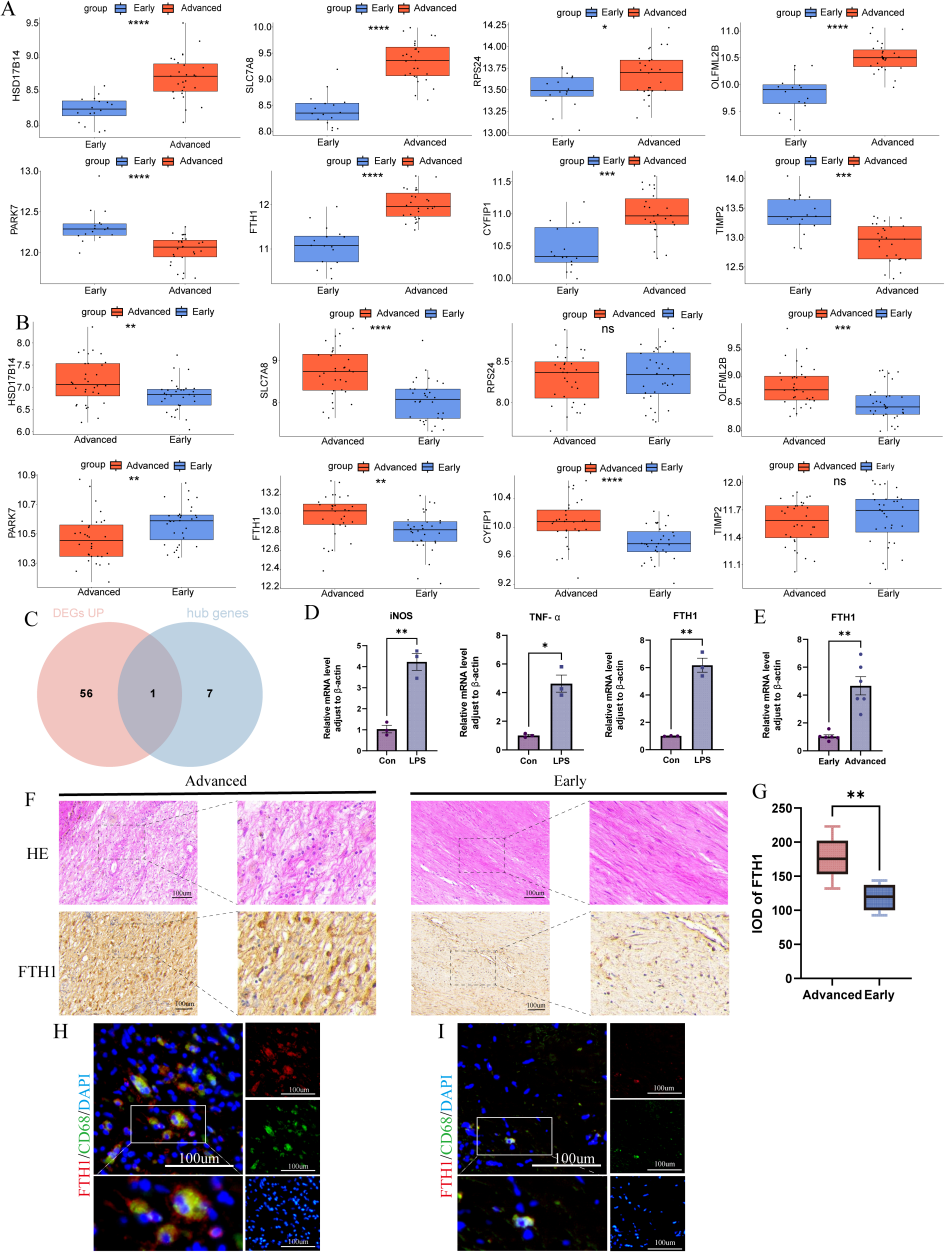
Verification of marker genes. (A) Expression levels of hub genes in the training dataset. The Wilcoxon test is employed to compare the two data sets. *p < 0.05; ***p < 0.001; ^****^ *p* < 0.0001. (B) Expression levels of hub genes in external validation dataset. The Wilcoxon test is employed to compare the two data sets. **p < 0.01; ***p < 0.001; ^****^ *p* < 0.0001; ^ns^ *p* > 0.05. (C) Venn diagram representing the intersection between hub genes and upregulated genes in the AC mono. (D) mRNA levels of *FTH1* in M1 macrophages. The student's *t*‐test is employed to compare the two groups. *p < 0.05; **p < 0. 01. (E) mRNA levels of *FTH1* in human advanced plaques. The student's t‐test is employed to compare the two groups. **p < 0. 01. (F) IHC images of FTH1 in advanced‐ and early‐stage carotid plaques. (G) IOD of FTH1. The student's t‐test is employed to compare the two groups. **p < 0.01. (H, I) Immunofluorescence staining showed that FTH1 and CD68 co‐localised, and the number of macrophages with high expression of FTH1 increased in advanced plaques compared with early plaques.

The expression levels of the eight hub genes in the training dataset GSE163154 are shown in Figure 9A. To enhance the reliability of our results, we used the external dataset GSE43292 to validate the expression levels of the eight hub genes. As shown in Figure 9B, the eight hub genes exhibited consistent expression trends in early‐ and advanced‐stage CAS, similar to the training dataset. Using differential expression analysis between AC and Other mono, we identified 57 upregulated genes in AC mono. Taking the intersection with the hub genes, we obtained *FTH1* for further research (Figure 9C). RT‐qPCR results revealed a high expression level of *FTH1* in M1 macrophages and plaque tissue, which is also consistent with the above analysis results (Figure 9D,E). As shown in Figure 9F, FTH1 protein expression levels were relatively high in advanced carotid plaques. Subsequent quantitative analysis indicated a statistically significant difference in the expression levels of the FTH1 (*p* = 0.0034) gene between early and advanced plaques (Figure 9G). Additionaly, immunofluorescence staining showed that FTH1 and CD68 co‐localised, and the number of macrophages with high expression of FTH1 increased in advanced plaques compared with early plaques (Figure 9H,I).

## 4 Discussion

Advanced plaque rupture and subsequent thrombosis formation‐induced cerebrovascular events pose a critical challenge to global public health. Early screening, prevention, and management in the progression of carotid atherosclerosis are crucial for reducing the incidence and disability rates of cerebrovascular events and improving outcomes [33]. Screening potential susceptibility genes and unravelling their biological mechanisms in carotid atherosclerosis progression can provide proven strategies for the predictive screening, targeted prevention, and personalised management of the disease. Therefore, in the present study, comprehensive mining of single‐cell sequencing data and transcriptome sequencing data combined with the LASSO regression algorithm identified *PARK7*, *RPS24*, *FTH1*, *SLC7A8*, *CYFIP1*, *OLFML2B*, *HSD17B14*, and *TIMP2* as novel features that participate in carotid atherosclerosis progression through the regulation of monocyte function and provide fresh strategies for the prevention and management of advanced carotid atherosclerosis.

The development of multi‐omics technologies has revolutionised healthcare for patients with carotid plaques, facilitating a paradigm shift from responsive to prophylactic treatment [34, 35, 36]. scRNA‐seq technology has rapidly developed in recent years, allowing for the identification of complex and rare cell populations and the discovery of regulatory relationships between genes [37], offering new possibilities for addressing biological and medical enquiries [38]. scRNA‐seq technology was first used in oncology and, more recently, in carotid plaques [39, 40]. Therefore, we analysed the scRNA‐seq data to reveal changes in different cell subpopulations and their proportions during plaque progression. We identified six cell clusters in carotid plaques: T cells, endothelial cells, smooth muscle cells, B cells, monocytes, and mast cells. Of these, the proportion of monocytes showed the most significant changes during plaque progression. We further divided monocyte subpopulations and identified five small subpopulations specific to advanced plaques. These five subpopulations were named AC mono and its functions were further explored. Cell–cell communication analysis revealed that AC mono had a higher level of interaction with other cell clusters, contributing to the progression of carotid plaque. In our results, AC mono facilitated the interactions with SMC through SPP1‐ITGB1 ligand–receptor pairs, which could lead to SMC phenotype switching and foam cell formation [41, 42], contributing to the progression of carotid plaque. Besides, in advanced carotid plaque, AC mono can interact with T cells through SPP1‐CD44, thereby inducing Th17 cell differentiation, and enhancing the inflammatory cascade reaction [43]. Of course, AC mono also interacted with many other cells that might be responsible for plaque progression, which required further research to explore. Genes highly associated with these subclusters were identified using hdWGCNA. According to Pseudotime analysis, the change of carotid plaque instability was accompanied by a transformation of monocyte–macrophage function. Macrophages present in early atherosclerotic plaques originate primarily from recruited monocytes, whereas macrophages proliferate in advanced plaques and their functions are affected by microenvironmental signals [44]. Intraplaque haemorrhage in advanced CASs stimulates macrophage polarisation [45], leading to an increased risk of plaque instability and rupture [46]. When haemorrhage occurs, released erythrocytes undergo clearance by macrophages, resulting in increased iron content and release of haem associated with lipids oxidisation [47]. Iron overload leads to uncontrolled inflammatory activation of macrophages, which is detrimental to plaque repair [48]. Our heatmap showed that genes related to iron metabolism and pro‐inflammatory phenotypes such as FTH1 and PKM were highly expressed in AC mono, whereas anti‐inflammatory phenotype markers such as CD163 were expressed more significantly in other mono. The macrophage phenotype with high expression of CD163 can produce anti‐inflammatory factors and clear haemoglobin–haptoglobin complexes through endocytosis, thereby preventing oxidative damage mediated by the accumulation of free haemoglobin and preventing plaque progression during intra‐plaque haemorrhage [49]. In addition, these macrophages can also contribute in inhibiting the accumulation of lipids and formation of foam cells [50]. Our Pseudotime results may contribute to identify potential intervention targets that influence the transformation of monocyte phenotypes, thereby interrupting or reversing plaque progression. Functional enrichment analysis revealed a shift in the metabolic functions of monocytes, including hypoxia, glycolysis, cholesterol homeostasis, and fatty acid metabolism. Hypoxia promotes the survival of monocytes and macrophages and alters their functions [51]. Furthermore, the hypoxic environment in plaques contributes to the stabilisation of hypoxia‐inducible factor‐1α (HIF‐1α), leading to plaque instability and increased levels of glycolysis in macrophage [52]. The pro‐inflammatory phenotype of macrophage is primarily driven by glycolysis, in which levels are significantly increased in rupture‐prone atherosclerotic plaque [53]. An imbalance in cholesterol homeostasis in macrophages accelerates cholesterol accumulation in lysosomes, leading to the formation of cholesterol crystals [54], the presence of which is a sign of plaque vulnerability [55]. Fatty acid metabolism also influences macrophage function, with oxidation linked to anti‐inflammatory phenotypes and synthesis associated with pro‐inflammatory phenotypes [56]. Besides, high levels of free fatty acids were shown to induce inflammation in macrophage, ultimately driving plaque instability [57, 58]. These changes lead to the formation of M1 macrophages and foam cells [59, 60], both of which are involved in plaque genesis and development [61].

The emerging machine‐learning algorithm LASSO has contributed to the identification of disease biomarkers and has revolutionised disease diagnosis [62]. The novelty of this study is that we identified key biomarkers associated with plaque progression through LASSO analysis, and subsequently combined multiple machine‐learning algorithms for predictive model construction. Among multiple machine‐learning models, RF model constructed using LASSO showed the highest diagnostic efficacy. Besides that, RF model has a strong capability to handle high‐dimensional data. By integrating multiple decision trees, it enhances prediction accuracy and stability while effectively reducing the risk of overfitting. Compared to the Support Vector Machine model, RF is more stable and does not require complex parameter tuning. In contrast to linear models, it could handle nonlinear relationships among complex data features. More importantly, RF can evaluate feature importance, thereby improving the interpretability of the model results [63]. Based on these properties and its outstanding performance in our results, the RF model was selected. We validated the predictive model using an independent external dataset, which facilitated the control of risk bias. The AUC value was 0.899, indicating an excellent diagnostic effect. A prediction model was then constructed based on the ratio of the expression level of LASSO genes to the expression level of immune cells using CNN, which is one of the most significant networks in the deep learning field widely applied in disease prediction and diagnosis [64]. However, studies applying CNN to construct predictive models for the progression of carotid plaques are lacking. This study presents a novel approach to predicting carotid plaque progression by creating a unique “portrait” of each patient using the ratio of LASSO gene to immune cell expression for inverse CNN building models for predicting carotid plaque progression. After 500 model training iterations, the AUC value of the training set of the CNN prediction model was 0.979, and the AUC value of the external validation set was 0.933, demonstrating superior diagnostic performance compared to machine learning. Collectively, this pioneering study provides new methods for constructing prediction models that could assist in preventing carotid plaque progression.

In the present study, *PARK7*, *RPS24*, *SLC7A8*, *CYFIP1*, *OLFML2B*, *HSD17B14*, *TIMP2*, and *FTH1* were identified as key markers of the pathogenesis and progression of carotid plaques. *PARK7* encodes proteins that belong to the peptidase C56 family and function as sensors of oxidative stress. It plays an important role in protecting cells against oxidative stress and cell death [65]. *PARK7* is involved in blocking metabolite and protein damage caused by glycolytic metabolites [66] and suppresses ferroptosis [67]. *PARK7* is required for the maintenance of mitochondrial morphology, function, and clearance of dysfunctional mitochondria [68]. A recent study found that *PARK7* deficiency leads to an imbalance in regulatory T cells (Treg) levels [69]. Tregs are engaged in maintaining immune homeostasis and preventing autoimmunity, including activating anti‐inflammatory macrophages, suppressing foam cell formation, and affecting cholesterol metabolism, all of which can prevent carotid atherosclerosis plaque progression [70]. This finding suggests that *PARK7* deficiency is involved in disease development by affecting metabolic homeostasis. *RPS24* encodes a ribosomal protein component of the 40S subunit. *RPS24* is closely associated with pathways related to cell proliferation and immune Infiltration [71]. Lysosomes control inflammation via cholesterol partitioning [72]. Overexpression of *RPS24* can affect ribosomal function and, thus, metabolic processes, leading to lipid accumulation and inflammation. All of these factors contribute to the onset and development of carotid plaques. *SLC7A8* encodes a protein belonging to the L‐type amino acid transporter (LAT) family that is responsible for transporting neutral amino acids into cells [73]. *SLC7A8* overexpression significantly enhances the cellular uptake of leucine and glutamine, which are two potent stimulators of rapamycin complex 1 (mTORC1) [74]. Activation of mTORC1 further induces HIF‐1α production, which promotes glycolysis and causes macrophages to differentiate into the pro‐inflammatory M1 type [75]. Mitotic inhibition, dysfunctional mitochondrial accumulation, and mitochondrial apoptosis induced by mTORC1 activation exacerbate macrophage apoptosis, which is believed to contribute to the progression of atherosclerotic plaques [76]. The protein encoded by *CYFIP1* promotes actin polymerisation to regulate cytoskeletal dynamics [77]. Additionally, the protein interacts with translation initiation factor 4E to inhibit protein translation [78]. Previous studies have found that *CYFIP1* overexpression leads to specific cellular phenotypes through the regulation of mTOR signalling [79]. In addition, *CYFIP1* may act as a MyD88‐dependent intermediate protein involved in the reorganisation of actin and cytoskeleton to regulate macrophage phagocytosis [80]. *OLFML2B* encodes an extracellular matrix protein [81]. There are limited studies on this gene, with most focusing on cancer. Our analysis showed that this gene was associated with many immune cells. We hypothesised that the overexpression of OLFML2B promotes tissue infiltration of inflammatory cells and accelerates carotid plaque progression. Further studies are needed to verify our hypotheses. Proteins of the 17‐beta‐hydroxysteroid dehydrogenase family, such as *HSD17B14*, primarily metabolise steroids and other substrates, such as fatty acids, prostaglandins, and xenobiotics [82]. Nicotinamide adenine dinucleotide (NAD) regulates numerous biochemical processes. “NAD+” and “NADH” are the oxidised and reduced forms of NAD, respectively. The NAD^+^/NADH ratio is essential for regulating the energy metabolism [83]. In vitro, *HSD17B14* converts NAD^+^ to NADH [82]. A decrease in NAD^+^ level is considered a major risk factor for atherosclerosis. Increasing NAD+ levels using various methods has a positive effect on halting carotid plaque development [84]. These studies suggest that HSD17B14 regulates carotid plaque development by affecting NAD^+^ levels. Proteins of the TIMP family are natural inhibitors of MMPs, which degrade the extracellular matrix [85]. High *TIMP2* expression reduces monocyte/macrophage infiltration into atherosclerotic lesions, protecting against atherosclerotic plaque progression and instability [86]. Our study found that TIMP2 levels were higher in early than in progressive plaque tissues, which is consistent with current findings. *FTH1* encodes the heavy subunit of ferritin, which can affect the rates of iron uptake and release in different tissues and cells by altering ferritin subunit composition [87]. Oxidised low‐density lipoprotein treatment of monocytes causes distinct overexpression of *FTH1* [88]. Neutrophils with high *FTH1* expression not only release more inflammatory factors when regulated by interleukin‐10, but also possess stronger antioxidant capacity and are less prone to apoptosis [89]. This leads to prolonged retention of neutrophils in tissues and aggravates inflammatory damage. In this study, we reached the same conclusion that *FTH1* expression increased in advanced carotid plaques. Additionally, the results of immune correlation analysis demonstrated that *FTH1* was primarily associated with monocytes and neutrophils. Analysis of upregulated genes in the AC mono and their intersection with hub genes revealed that FTH1 influences monocyte/macrophage function and plays an important role in progressive carotid plaques. In our study, *FTH1* was significantly elevated in advanced carotid plaques and M1 macrophages, as confirmed by PCR and immunohistochemistry, further corroborating our conclusion. Previous studies have revealed that *FTH1* is highly expressed in M1 macrophages and is engaged in regulating macrophage polarisation and function [90]. Other studies have reported that *FTH1*, a suppressor of ferroptosis, can reduce free iron levels [91]. Here, we hypothesised that an increase in *FTH1* may serve as a defence mechanism against ferroptosis in M1 macrophages. This may lead to the long‐term survival of pro‐inflammatory macrophages in the plaque tissue. However, low FTH1‐expressing M2 macrophages are unable to exert anti‐inflammatory and reparative effects due to the onset of ferroptosis. An imbalance between tissue damage and repair accelerates plaque progression and increases the risk of plaque rupture.

Ferritin, a cellular iron storage protein, consists of ferritin‐H (encoded by FTH1) and ferritin‐L (encoded by FTL), and it can be released via the exosome pathway [92, 93]. The monocytes with high FTH1 expression identified in our study may release ferritin into the serum through this pathway. Notably, Wolff et al. found a positive relationship between serum ferritin levels and the incidence of carotid plaques [94], while the relationship between ferritin levels and plaque stability remains unclear. The markers we identified offer a novel perspective for distinguishing between early and advanced carotid plaques using serum markers. Furthermore, monocytes/macrophages and neutrophils with high FTH1 expression demonstrate enhanced pro‐inflammatory, antioxidant, and anti‐apoptotic capabilities [89, 90, 95], allowing them to persist in plaque tissues and inflict ongoing inflammatory damage. This makes FTH1 a potential therapeutic target. Targeting FTH1 with specific inhibitors may alter the levels and functions of inflammatory cells within plaque tissue, potentially preventing plaque progression.

Furthermore, differences in the immune landscape between advanced‐ and early‐stage CAS were visualised using single‐cell analysis with UMAP. Advanced CAS show significantly higher levels of immune cell infiltration, particularly monocytes compared to early atherosclerotic plaques. In atherosclerosis, monocytes and macrophages are recruited to the matrix beneath the endothelial cell layer, and these cells are involved in the inflammatory response and formation of the necrotic core [96]. The balance of macrophages in plaques is dynamic, and alterations in the quantity and function of macrophages affect the fate of plaques [97]. Recent findings have highlighted the significance of metabolic and functional reprogramming of monocytes and macrophages in the progression of atherosclerosis [56]. This is consistent with our finding that advanced plaques contain a specific subpopulation of monocytes which possess a more enriched metabolic process. In addition, we analysed the correlation between immune cells and key markers to further investigate the regulation of different immune cell functions. Our results showed that the expression of *CYFIP1*, *FTH1*, *OLFML2B*, *SLC7A8*, and *HSD17B14* was positively correlated with monocytic lineage expression in progressive plaque tissues, whereas *TIMP2* was negatively correlated with monocytic lineage expression. Since we extracted key markers from key modular genes of monocyte subpopulations, these results are consistent with our expectations.

Based on the eight key markers, we further subdivided the advanced plaque tissue into two subtypes, subtype 1 and subtype 2. We then performed monocyte scoring for both subtypes based on the expression levels of the key module genes. The results showed a difference in monocyte scores between the two subtypes, with subtype 1 having higher scores than those of subtype 2. Likewise, our findings of immune infiltration analysis suggest a more pronounced immune infiltration in subtype 1, especially the monocyte lineage and neutrophil lineage. In the case of monocyte lineages, their spatial distribution within atherosclerotic plaques differs, with pro‐inflammatory monocytes/macrophages being enriched in more unstable and rupture‐prone areas [98]. Furthermore, monocytes can differentiate into macrophages, facilitating the secretion of inflammatory factors, the production of connective tissue, and the formation of fibrous caps [99, 100]. Several studies suggest that thin‐cap atheroma accompanied with the presence of abundant macrophages within the cap likely associated with high risk of plaque rupture [101, 102]. For neutrophil lineages, the count of neutrophil has been shown to correlate with atherosclerosis severity and plaque instability [103]. Neutrophils can release various proteases, including MMPs, which can not only lead to the degradation of elastic fibres but also cause thinning of the fibrous cap and plaque erosion [104]. In addition, a recent study has also found that neutrophil extracellular traps, released by neutrophils, significantly increase carotid plaque instability [105]. Therefore, subtype 1, which has higher monocyte and neutrophil infiltration, may with more rapid plaque progression and an accompanying poorer prognosis than subtype 2. The primary prevention of atherosclerosis is dependent on the effective reduction of risk factors, including smoking control, increased physical exercise, a healthier diet, weight loss, prevention, and treatment of hypertension and diabetes [106]. From the perspective of early intervention, a comprehensive evaluation should be conducted on healthy individuals with abnormal expression of the eight key biomarkers and patients with early carotid plaques, actively controlling risk factors to prevent or delay the onset and development of carotid plaques. For advanced plaques, there were difference in the degree of immune infiltration between different subtype, which might lead to difference in the risk of rupture and the option of treatment [107, 108]. Therefore, it is necessary to measure the expression levels of eight key markers to further classify patients with advanced carotid plaques, which will facilitate personalised patient care. The higher the expression levels of the key genes, the earlier the initiation of secondary and tertiary prevention is recommended to prevent further progression and poor prognosis. Overall, the eight key biomarkers we screened can be used not only to identify early and progressive plaques but also to stratify the risk of patients with progressive plaques, which is key to personalised medicine for patients with carotid atherosclerosis.

Although our analysis combined with machine‐learning techniques provides novel insights of the progression of carotid plaque, there are still limitations. Firstly, the source of a single data set or insufficient sample size may restrict the generalizability of our results. Secondly, due to the lack of sufficient in vivo and in vitro experimental data to fully verify the functions and mechanisms of these markers, our conclusions may not be comprehensive enough. For future studies, larger‐scale data, deeper functional experiments, and more advanced omics analysis are indispensable for a more comprehensive understanding of carotid plaque progression.

## 5 Conclusions

In conclusion, our study revealed differences in the immune landscapes of carotid plaques over time. Furthermore, *PARK7*, *RPS24*, *FTH1*, *SLC7A8*, *CYFIP1*, *OLFML2B*, *HSD17B14*, and *TIMP2* were identified as key markers associated with advanced carotid atherosclerosis. In addition, our study established promising machine‐learning and deep learning models that can guide the development of targeted prevention and personalised treatment for advanced carotid atherosclerosis.

## Author Contributions

**Han Zhang:** conceptualization (lead), data curation (lead), formal analysis (lead), methodology (lead), software (lead), visualization (lead), writing – original draft (lead), writing – review and editing (equal). **Yixian Wang:** conceptualization (equal), methodology (equal), software (equal). **Mingyu Liu:** data curation (equal), formal analysis (equal), software (equal). **Yao Qi:** methodology (equal), software (equal), validation (equal). **Shikai Shen:** data curation (equal), formal analysis (equal), methodology (equal). **Qingwei Gang:** data curation (equal), methodology (equal), validation (equal). **Han Jiang:** supervision (equal), validation (equal), writing – original draft (supporting). **Yu Lun:** validation (equal), writing – original draft (supporting), writing – review and editing (supporting). **Jian Zhang:** conceptualization (equal), supervision (equal), writing – original draft (supporting), writing – review and editing (equal).

## Conflicts of Interest

The authors declare no conflicts of interest.

## Supporting information


Data S1.

## Data Availability

The data are public and can be downloaded from the GEO database.
