# Supplementary material for: Deep Learning and Single‐Cell Sequencing Analyses Unveiling Key Molecular Features in the Progression of Carotid Atherosclerotic Plaque
Source: J Cell Mol Med. 2024 Nov 25;28(22):e70220. doi: 10.1111/jcmm.70220 (PMC11588433; doi:10.1111/jcmm.70220)
Supplement: Supplementary file 1 — Data S1. [file JCMM-28-e70220-s001.docx]

**S1: Cell markers for annotation and proofreading**

T cells (CD3D, CD3E, IL7R and NKG7)

B cells (CD79A and MS4A1)

Smooth muscle cell (MYH11, ACTA2)

Monocyte (FCN1, CD68, LYZ, S100A9, C1QAand CD14)

Endothelial cells (VWF and PECAM1)

Mast cells (CPA3 and KIT)

**S2: Construction of Convolutional Neural Networks (CNNs)**

1. Adjust input data to compatible with CNN input requirements
   1. The "IOBR::deconvo_mcpcounter" function was used to evaluated 10 types of immune cells in the test data set GSE163154 and training data set GSE28829 samples.
   2. Immune cell composition data were then combined with gene expression data to create a data format suitable for machine learning model training, which was then visualized by heat maps.
   3. The heat map constructed by the ratio of central gene expression levels to immune cell expression levels.
2. Deep Learning model construction
   1. The CNN model was constructed based on Keras and TensorFlow and did not use cross-validation technology. Firstly, create a sequential model using the “keras_model_sequential” function.
   2. A first 2D convolutional layer is added, which uses 32 filters (kernels) with a kernel size of 3x3. Use "same" padding to ensure the output dimensions are the same as the input dimensions. Add a "ReLU" activation function after the convolutional layer to increase nonlinearity.
   3. Add a second convolutional layer and a pooling layer. Different from the first layer, the second had 16 filters with a convolution kernel size of 2 × 2. Also use "same" for padding. No dilated convolution is performed and use "Softplus" activation function.
   4. A maximum pooling layer is added, and the pooling window is 2x2 in size.
   5. Add a flattening layer to flatten the multi-dimensional input into one dimension for input to the fully connected layer.
   6. Construct a fully connected layer containing 64 neurons, using "ReLU" as the activation function. A dropout layer is then added to drop 50% of the neurons to prevent overfitting.
   7. An output layer containing 1 neuron is added and using Sigmoid as the activation function for binary classification tasks.
   8. Using binary cross-entropy as the loss function, Adam as the optimizer, and accuracy as the evaluation metric to compile the model.
   9. After the model was constructed, it was trained for 500 times, and the loss and accuracy curves during the training process are plotted.
   10. Finally, the trained model was used to predict the training set and test set, and the ROC curve was drawn to evaluate the model performance

**S3: Expression information for the 57 upregulated genes in AC mono**

| gene | p_val | avg_log2FC | pct.1 | pct.2 | p_val_adj | cluster |
| --- | --- | --- | --- | --- | --- | --- |
| SPP1 | 0 | 3.334671 | 0.953 | 0.343 | 0 | AC_Mono |
| FABP5 | 0 | 3.218898 | 0.976 | 0.558 | 0 | AC_Mono |
| CSTB | 0 | 2.831985 | 0.993 | 0.81 | 0 | AC_Mono |
| CCL7 | 0 | 2.405382 | 0.47 | 0.031 | 0 | AC_Mono |
| FN1 | 0 | 2.287274 | 0.734 | 0.185 | 0 | AC_Mono |
| CTSL | 0 | 2.241228 | 0.946 | 0.596 | 0 | AC_Mono |
| TIMP1 | 0 | 2.14781 | 0.968 | 0.742 | 0 | AC_Mono |
| FBP1 | 0 | 2.101356 | 0.827 | 0.333 | 0 | AC_Mono |
| C15orf48 | 0 | 1.936995 | 0.912 | 0.319 | 0 | AC_Mono |
| MARCO | 0 | 1.922599 | 0.823 | 0.224 | 0 | AC_Mono |
| CTSD | 0 | 1.862019 | 0.974 | 0.777 | 0 | AC_Mono |
| PLIN2 | 0 | 1.728993 | 0.956 | 0.605 | 0 | AC_Mono |
| RGCC | 0 | 1.713493 | 0.928 | 0.485 | 0 | AC_Mono |
| S100A10 | 0 | 1.709497 | 0.999 | 0.863 | 0 | AC_Mono |
| GAPDH | 0 | 1.679593 | 0.999 | 0.948 | 0 | AC_Mono |
| ENO1 | 0 | 1.644822 | 0.976 | 0.698 | 0 | AC_Mono |
| VIM | 0 | 1.638259 | 1 | 0.946 | 0 | AC_Mono |
| PHLDA1 | 0 | 1.617174 | 0.85 | 0.258 | 0 | AC_Mono |
| TPI1 | 0 | 1.60551 | 0.977 | 0.788 | 0 | AC_Mono |
| SLAMF9 | 0 | 1.571528 | 0.622 | 0.041 | 0 | AC_Mono |
| MIF | 0 | 1.538193 | 0.922 | 0.463 | 0 | AC_Mono |
| SDC2 | 0 | 1.523946 | 0.707 | 0.136 | 0 | AC_Mono |
| PGK1 | 0 | 1.514969 | 0.946 | 0.748 | 0 | AC_Mono |
| GPNMB | 0 | 1.480125 | 0.87 | 0.26 | 0 | AC_Mono |
| LDHA | 0 | 1.411894 | 0.961 | 0.717 | 0 | AC_Mono |
| LGALS1 | 0 | 1.410035 | 0.991 | 0.798 | 0 | AC_Mono |
| CD52 | 0 | 1.344285 | 0.904 | 0.364 | 0 | AC_Mono |
| SH3BGRL3 | 0 | 1.243907 | 0.996 | 0.925 | 0 | AC_Mono |
| PKM | 0 | 1.219639 | 0.949 | 0.642 | 0 | AC_Mono |
| MMP19 | 0 | 1.139828 | 0.628 | 0.116 | 0 | AC_Mono |
| FTH1 | 0 | 1.042979 | 1 | 0.999 | 0 | AC_Mono |
| ERO1A | 3.96E-289 | 1.262901 | 0.699 | 0.22 | 1.33E-284 | AC_Mono |
| FTL | 2.09E-288 | 1.138587 | 1 | 0.997 | 7.02E-284 | AC_Mono |
| CD36 | 8.69E-286 | 1.994201 | 0.834 | 0.422 | 2.91E-281 | AC_Mono |
| GSTO1 | 2.42E-282 | 1.160819 | 0.951 | 0.681 | 8.10E-278 | AC_Mono |
| CD63 | 1.24E-276 | 1.060425 | 0.995 | 0.898 | 4.15E-272 | AC_Mono |
| FABP4 | 2.65E-274 | 3.733251 | 0.518 | 0.101 | 8.90E-270 | AC_Mono |
| CD68 | 8.69E-269 | 1.114464 | 0.975 | 0.831 | 2.91E-264 | AC_Mono |
| CYP27A1 | 2.31E-267 | 1.098513 | 0.561 | 0.122 | 7.74E-263 | AC_Mono |
| PPP1R14B | 1.15E-249 | 1.030171 | 0.865 | 0.478 | 3.87E-245 | AC_Mono |
| ATP6V1F | 6.09E-249 | 1.041817 | 0.951 | 0.754 | 2.04E-244 | AC_Mono |
| P4HA1 | 9.05E-245 | 1.245455 | 0.714 | 0.262 | 3.03E-240 | AC_Mono |
| CTSB | 3.58E-233 | 1.201826 | 0.978 | 0.845 | 1.20E-228 | AC_Mono |
| CCL2 | 1.20E-231 | 2.411297 | 0.776 | 0.373 | 4.03E-227 | AC_Mono |
| H2AFY | 3.30E-228 | 1.012181 | 0.923 | 0.693 | 1.11E-223 | AC_Mono |
| TUBA1C | 4.30E-212 | 1.015881 | 0.848 | 0.475 | 1.44E-207 | AC_Mono |
| IL1RN | 2.63E-197 | 1.471676 | 0.8 | 0.381 | 8.82E-193 | AC_Mono |
| VCAN | 7.62E-193 | 1.186165 | 0.85 | 0.414 | 2.55E-188 | AC_Mono |
| RNASE1 | 2.31E-192 | 1.21081 | 0.79 | 0.374 | 7.74E-188 | AC_Mono |
| MT1G | 3.68E-192 | 2.85085 | 0.289 | 0.028 | 1.24E-187 | AC_Mono |
| APOC1 | 3.84E-191 | 1.819566 | 0.715 | 0.31 | 1.29E-186 | AC_Mono |
| LGALS3 | 4.81E-174 | 1.505142 | 0.936 | 0.75 | 1.61E-169 | AC_Mono |
| MT1X | 5.80E-151 | 2.398865 | 0.504 | 0.189 | 1.95E-146 | AC_Mono |
| FAM162A | 4.98E-146 | 1.059836 | 0.633 | 0.31 | 1.67E-141 | AC_Mono |
| MT1H | 1.83E-107 | 1.922993 | 0.145 | 0.009 | 6.14E-103 | AC_Mono |
| MT2A | 1.18E-96 | 2.173488 | 0.699 | 0.458 | 3.96E-92 | AC_Mono |
| CXCL5 | 1.55E-71 | 1.060344 | 0.129 | 0.017 | 5.21E-67 | AC_Mono |

**S4: Primer sequences (5'-3')**

β-actin-mus (forward: 5'-TTCCAGCCTTCCTTCTTG-3',

reverse: 5'-GGAGCCAGAGCAGTAATC-3'),

FTH1-mus (forward: 5'-CAAGTGCGCCAGAACTACCA-3',

reverse: 5'-GCCACATCATCTCGGTCAAAA-3'),

iNOS-mus (forward: 5'- TGCCAGGGTCACAACTTTACA-3',

reverse: 5'- CAGCTCAGTCCCTTCACCAA-3'),

TNF-α-mus (forward: 5'- GATCGGTCCCCAAAGGGATG-3',

reverse: 5'- GTTTGCTACGACGTGGGCT-3'),

β-actin-homo (forward: 5'-CATGTACGTTGCTATCCAGGC-3',

reverse: 5'-CTCCTTAATGTCACGCACGAT-3'),

FTH1-homo (forward:5'-GACCCCCATTTGTGTGACTTC-3',

reverse: 5'- ATTATCACTGTCTCCCAGGGT-3').
